# Supplementary material for: Cost-effectiveness of mandatory folic acid fortification of flours in prevention of neural tube defects: A systematic review
Source: PLoS One. 2021 Oct 21;16(10):e0258488. doi: 10.1371/journal.pone.0258488 (PMC8530293; doi:10.1371/journal.pone.0258488)
Supplement: S2 File — (DOCX) [file pone.0258488.s002.docx]

**Electronic Search Strategy**

The following is the electronic search terms we used in MEDLINE/PubMed, Web of Science, Scopus, Embase, EBSCO/CINAHL:

("mandatory" OR "mandatory flour" OR "mandatory fa" OR "mandatory flour fortification" OR "mandatory fa fortification" OR "mandatory folate fortification" OR "mandatory flour fortification program" OR "mandatory folic acid" OR "mandatory food fortification" OR "mandatory folic acid food" OR "mandatory folic acid fortification" OR "folic acid flour fortification" OR "folic acid food" OR "folic acid flour" OR "folic acid food fortification" OR "folic acid fortification" OR "folic acid food fortification program" OR "folic acid fortification policy" OR "folic acid fortification policies" OR "folic acid fortification programs" OR "folic acid fortified" OR "folic acid fortification program" OR "folic acid fortified bread" OR "folic acid oral supplementation" OR "folic acid fortified cereal grain" OR "enriched bread" OR "enriched breads" OR "enriched breakfast" OR "enriched breakfast cereals" OR "enriched corn" OR "enriched grain" OR "enriched food" OR "enriched foods" OR "enriched grain products" OR "enriched grains" OR "rich food" OR "rich foods" OR "vitamin supplement" OR "vitamin supplementation" OR "vitamin supplemented") AND ("economic evolution" OR "cost analysis" OR "cost effectiveness" OR "cost effectiveness analysis" OR "cea" OR "cost effectiveness analyses" OR "cost benefit" OR "cost benefit analysis" OR "cost benefit analyses" OR "cba" OR "cost saving" OR "cost savings analysis" OR "cost savings analyses" OR "cost consequence" OR "cost consequence analysis" OR "cost consequence analyses" OR "cca" OR "cost utility" OR "cost utility analysis" OR "cost utility analyses" OR "cua" OR "cost minimization" OR "cost minimization analysis" OR "cost minimization analyses" OR "cma").

This search strategy can be described more clearly in English as follows. The two numbered items below were combined using the AND command in Boolean logic.

1. Search for terms in the title or abstract related with intervention folic acid food fortification (flour, grains, cereal, enrich food) and vitamin supplements:

- 1. Mandatory flour OR Mandatory flour fortification OR Mandatory folic acid OR Mandatory fa OR Mandatory fa fortification OR Mandatory folate fortification OR Mandatory flour fortification program OR Mandatory food fortification OR Mandatory folic acid food **OR**
  2. Folic acid flour fortification OR Folic acid food OR Folic acid flour OR Folic acid food fortification OR Folic acid fortification OR Folic acid food fortification program OR Folic acid fortification programs OR Folic acid fortification policy OR Folic acid fortification policies OR folic acid fortified OR folic acid fortified bread OR Folic acid fortified cereal grain **OR**
  3. Enriched bread OR Enriched breads OR Enriched breakfast OR Enriched breakfast cereals OR Enriched corn OR Enriched grain OR Enriched food OR Enriched foods OR Enriched grain products OR Enriched grains OR Rich food" OR Rich foods **OR**
  4. Vitamin supplement OR Vitamin supplementation OR Vitamin supplemented OR Folic acid supplement.

1. **AND** search for terms in the title related to economic analysis:
   1. Economic evolution OR Cost analysis **OR**
   2. Cost effectiveness OR Cost effectiveness analysis” OR Cost effectiveness analyses OR cea **OR**
   3. Cost benefit OR Cost benefit analysis OR Cost benefit analyses OR cba **OR**
   4. Cost saving OR Cost savings analysis OR Cost savings analyses **OR**
   5. Cost consequence OR "cost consequence analysis" OR Cost consequence analyses OR cca **OR**
   6. Cost utility OR Cost utility analysis OR Cost utility analyses OR cua **OR**
   7. Cost minimization OR Cost minimization analysis OR Cost minimization analyses OR cma.

**History and Search Details:**

1. **PUMED/MEDLINE**

**Date: 02/22/21**

**#1** results 64,898 ("mandatory" OR "mandatory flour" OR "mandatory fa" OR "mandatory flour fortification" OR "mandatory fa fortification" OR "mandatory folate fortification" OR "mandatory flour fortification program" OR "mandatory folic acid" OR "mandatory food fortification" OR "mandatory folic acid food" OR "mandatory folic acid fortification" OR "folic acid flour fortification" OR "folic acid food" OR "folic acid flour" OR "folic acid food fortification" OR "folic acid fortification" OR "folic acid food fortification program" OR "folic acid fortification policy" OR "folic acid fortification policies" OR "folic acid fortification programs" OR "folic acid fortified" OR "folic acid fortification program" OR "folic acid fortified bread" OR "folic acid oral supplementation" OR "folic acid fortified cereal grain" OR "enriched bread" OR "enriched breads" OR "enriched breakfast" OR "enriched breakfast cereals" OR "enriched corn" OR "enriched grain" OR "enriched food" OR "enriched foods" OR "enriched grain products" OR "enriched grains" OR "rich food" OR "rich foods" OR "vitamin supplement" OR "vitamin supplementation" OR "vitamin supplemented")

**# 2** results 261,978 ("economic evolution" OR "cost analysis" OR "cost effectiveness" OR "cost effectiveness analysis" OR "cea" OR "cost effectiveness analyses" OR "cost benefit" OR "cost benefit analysis" OR "cost benefit analyses" OR "cba" OR "cost saving" OR "cost savings analysis" OR "cost savings analyses" OR "cost consequence" OR "cost consequence analysis" OR "cost consequence analyses" OR "cca" OR "cost utility" OR "cost utility analysis" OR "cost utility analyses" OR "cua" OR "cost minimization" OR "cost minimization analysis" OR "cost minimization analyses" OR "cma")

**#3** results 1,062 **#1 AND #2**

1. **SCOPUS**

**Date 02/24/2021**

**#1** results 665,031 TITLE-ABS-KEY ( "economic evolution" OR "cost analysis" OR "cost effectiveness" OR "cost effectiveness analysis" OR "cea" OR "cost effectiveness analyses" OR "cost benefit" OR "cost benefit analysis" OR "cost benefit analyses" OR "cba" OR "cost saving" OR "cost savings analysis" OR "cost savings analyses" OR "cost consequence" OR "cost consequence analysis" OR "cost consequence analyses" OR "cca" OR "cost utility" OR "cost utility analysis" OR "cost utility analyses" OR "cua" OR "cost minimization" OR "cost minimization analysis" OR "cost minimization analyses" OR "cma" )

#**2** results 150,893 TITLE-ABS-KEY ( "mandatory" OR "mandatory flour" OR "mandatory fa" OR "mandatory flour fortification" OR "mandatory fa fortification" OR "mandatory folate fortification" OR "mandatory flour fortification program" OR "mandatory folic acid" OR "mandatory food fortification" OR "mandatory folic acid food" OR "mandatory folic acid fortification" OR "folic acid flour fortification" OR "folic acid food" OR "folic acid flour" OR "folic acid food fortification" OR "folic acid fortification" OR "folic acid food fortification program" OR "folic acid fortification policy" OR "folic acid fortification policies" OR "folic acid fortification programs" OR "folic acid fortified" OR "folic acid fortification program" OR "folic acid fortified bread" OR "folic acid oral supplementation" OR "folic acid fortified cereal grain" OR "enriched bread" OR "enriched breads" OR "enriched breakfast" OR "enriched breakfast cereals" OR "enriched corn" OR "enriched grain" OR "enriched food" OR "enriched foods" OR "enriched grain products" OR "enriched grains" OR "rich food" OR "rich foods" OR "vitamin supplement" OR "vitamin supplementation" OR "vitamin supplemented" )

**#3** results 2003 **#1 AND #2**

1. **WEB OF SCIENCE**

**Date. 02/24/2021**

**#1** results 77.296 TOPICO: ("mandatory" OR "mandatory flour" OR "mandatory fa" OR "mandatory flour fortification" OR "mandatory fa fortification" OR "mandatory folate fortification" OR "mandatory flour fortification program" OR "mandatory folic acid" OR "mandatory food fortification" OR "mandatory folic acid food" OR "mandatory folic acid fortification" OR "folic acid flour fortification" OR "folic acid food" OR "folic acid flour" OR "folic acid food fortification" OR "folic acid fortification" OR "folic acid food fortification program" OR "folic acid fortification policy" OR "folic acid fortification policies" OR "folic acid fortification programs" OR "folic acid fortified" OR "folic acid fortification program" OR "folic acid fortified bread" OR "folic acid oral supplementation" OR "folic acid fortified cereal grain" OR "enriched bread" OR "enriched breads" OR "enriched breakfast" OR "enriched breakfast cereals" OR "enriched corn" OR "enriched grain" OR "enriched food" OR "enriched foods" OR "enriched grain products" OR "enriched grains" OR "rich food" OR "rich foods" OR "vitamin supplement" OR "vitamin supplementation" OR "vitamin supplemented")

**#2** results 219.806 TOPICO ("economic evolution" OR "cost analysis" OR "cost effectiveness" OR "cost effectiveness analysis" OR "cea" OR "cost effectiveness analyses" OR "cost benefit" OR "cost benefit analysis" OR "cost benefit analyses" OR "cba" OR "cost saving" OR "cost savings analysis" OR "cost savings analyses" OR "cost consequence" OR "cost consequence analysis" OR "cost consequence analyses" OR "cca" OR "cost utility" OR "cost utility analysis" OR "cost utility analyses" OR "cua" OR "cost minimization" OR "cost minimization analysis" OR "cost minimization analyses" OR "cma")

**#3** results 748 **#1 AND #2**

1. **EBSCO/host - CINAHL**

**Date 02/24/2021**

**S1** results 67,042 "economic evolution" OR "cost analysis" OR "cost effectiveness" OR "cost effectiveness analysis" OR "cea" OR "cost effectiveness analyses" OR "cost benefit" OR "cost benefit analysis" OR "cost benefit analyses" OR "cba" OR "cost saving" OR "cost savings analysis" OR "cost savings analyses" OR "cost consequence" OR "cost consequence analysis" OR "cost consequence analyses" OR "cca" OR "cost utility" OR "cost utility analysis" OR "cost utility analyses" OR "cua" OR "cost minimization" OR "cost minimization analysis" OR "cost minimization analyses" OR "cma"

**S2** results 8,599 “mandatory" OR "mandatory flour" OR "mandatory fa" OR "mandatory flour fortification" OR "mandatory fa fortification" OR "mandatory folate fortification" OR "mandatory flour fortification program" OR "mandatory folic acid" OR "mandatory food fortification" OR "mandatory folic acid food" OR "mandatory folic acid fortification" OR "folic acid flour fortification" OR "folic acid food" OR "folic acid flour" OR "folic acid food fortification" OR "folic acid fortification" OR "folic acid food fortification program" OR "folic acid fortification policy" OR "folic acid fortification policies" OR "folic acid fortification programs" OR "folic acid fortified" OR "folic acid fortification program" OR "folic acid fortified bread" OR "folic acid oral supplementation" OR "folic acid fortified cereal grain" OR "enriched bread" OR "enriched breads" OR "enriched breakfast" OR "enriched breakfast cereals" OR "enriched corn" OR "enriched grain" OR "enriched food" OR "enriched foods" OR "enriched grain products" OR "enriched grains" OR "rich food" OR "rich foods" OR "vitamin supplement" OR "vitamin supplementation" OR "vitamin supplemented"

**S3** results 2,979 **S1 AND S2**

1. **EMBASE**

**Date 03/01/2021**

**#1** results 88,337 'mandatory':ti,ab,kw OR 'mandatory flour':ti,ab,kw OR 'mandatory fa':ti,ab,kw OR 'mandatory flour fortification':ti,ab,kw OR 'mandatory fa fortification':ti,ab,kw OR 'mandatory folate fortification':ti,ab,kw OR 'mandatory flour fortification program':ti,ab,kw OR 'mandatory folic acid':ti,ab,kw OR 'mandatory food fortification':ti,ab,kw OR 'mandatory folic acid food':ti,ab,kw OR 'mandatory folic acid fortification':ti,ab,kw OR 'folic acid flour fortification':ti,ab,kw OR 'folic acid food':ti,ab,kw OR 'folic acid flour':ti,ab,kw OR 'folic acid food fortification':ti,ab,kw OR 'folic acid fortification':ti,ab,kw OR 'folic acid food fortification program':ti,ab,kw OR 'folic acid fortification policy':ti,ab,kw OR 'folic acid fortification policies':ti,ab,kw OR 'folic acid fortification programs':ti,ab,kw OR 'folic acid fortified':ti,ab,kw OR 'folic acid fortification program':ti,ab,kw OR 'folic acid fortified bread':ti,ab,kw OR 'folic acid oral supplementation':ti,ab,kw OR 'folic acid fortified cereal grain':ti,ab,kw OR 'enriched bread':ti,ab,kw OR 'enriched breads':ti,ab,kw OR 'enriched breakfast':ti,ab,kw OR 'enriched breakfast cereals':ti,ab,kw OR 'enriched corn':ti,ab,kw OR 'enriched grain':ti,ab,kw OR 'enriched food':ti,ab,kw OR 'enriched foods':ti,ab,kw OR 'enriched grain products':ti,ab,kw OR 'enriched grains':ti,ab,kw OR 'rich food':ti,ab,kw OR 'rich foods':ti,ab,kw OR 'vitamin supplement':ti,ab,kw OR 'vitamin supplementation':ti,ab,kw OR 'vitamin supplemented':ti,ab,kw

**# 2** results 195,475 'economic evolution':ti,ab,kw OR 'cost analysis':ti,ab,kw OR 'cost effectiveness':ti,ab,kw OR 'cost effectiveness analysis':ti,ab,kw OR 'cea':ti,ab,kw OR 'cost effectiveness analyses':ti,ab,kw OR 'cost benefit':ti,ab,kw OR 'cost benefit analysis':ti,ab,kw OR 'cost benefit analyses':ti,ab,kw OR 'cba':ti,ab,kw OR 'cost saving':ti,ab,kw OR 'cost savings analysis':ti,ab,kw OR 'cost savings analyses':ti,ab,kw OR 'cost consequence':ti,ab,kw OR 'cost consequence analysis':ti,ab,kw OR 'cost consequence analyses':ti,ab,kw OR 'cca':ti,ab,kw OR 'cost utility':ti,ab,kw OR 'cost utility analysis':ti,ab,kw OR 'cost utility analyses':ti,ab,kw OR 'cua':ti,ab,kw OR 'cost minimization':ti,ab,kw OR 'cost minimization analysis':ti,ab,kw OR 'cost minimization analyses':ti,ab,kw OR 'cma':ti,ab,kw

**#3** results 1064 **#1 AND #2**


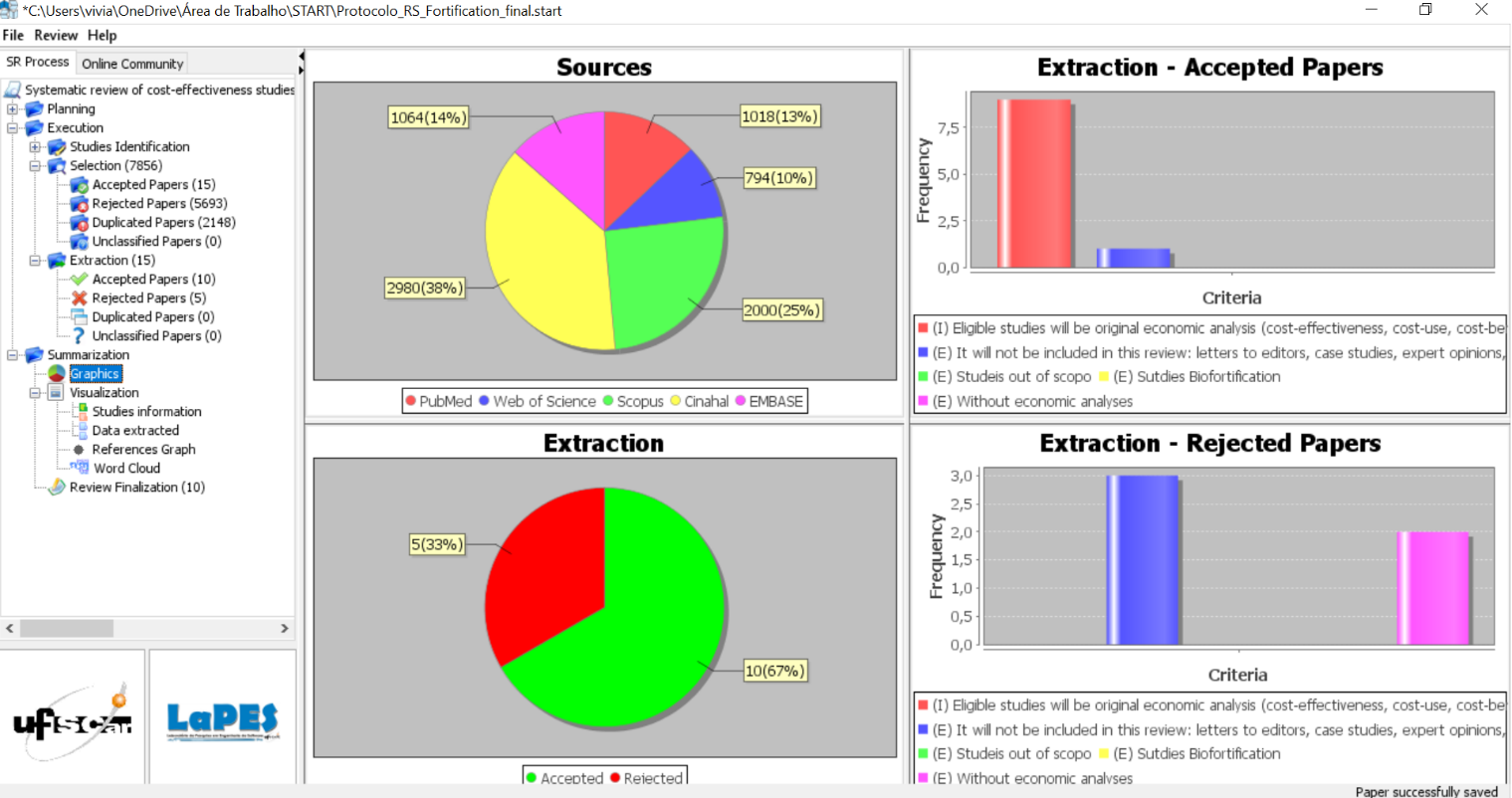
**Summary of selection articles**

From: screenshot of tool StArt (State of the Art through Systematic Review).
