# Supplementary material for: Cost-effectiveness of mandatory folic acid fortification of flours in prevention of neural tube defects: A systematic review
Source: PLoS One. 2021 Oct 21;16(10):e0258488. doi: 10.1371/journal.pone.0258488 (PMC8530293; doi:10.1371/journal.pone.0258488)
Supplement: S3 File — (DOCX) [file pone.0258488.s003.docx]

| Criteria | | Romano [23] | Bagriansky [24] | Grosse [25] | Grosse [33] | FSANZ [26] | FSANZ [34] | Llanos [27] | Jentink [29] | Sayed [28] | Bentley [30] | Dalziel [31] | Rabovskaja [32] | Saing [35] |
| --- | --- | --- | --- | --- | --- | --- | --- | --- | --- | --- | --- | --- | --- | --- |
| 1. Was a well-defined question posed in answerable form? | | Yes | Yes | Yes | Yes | Yes | Yes | Yes | Yes | Yes | Yes | Yes | Yes | Yes |
| 2. Was a comprehensive description of the competing alternatives given? (i.e., can you tell whom, where, and how often?) | | Yes | No | Yes | Yes | Yes | Yes | Yes | Yes | Yes | Yes | Yes | Yes | Yes |
| 3. Was the effectiveness of the programs or services established? | | Yes | No | Yes | Yes | No | Yes | No | No | No | No | Yes | Yes | No |
| 4. Were all the important and relevant costs and consequences for each alternative identified? | | No | No | No | Yes | Yes | Yes | No | Yes | No | No | No | Yes | Yes |
| 5. Were costs and consequences measured accurately inappropriate physical units (for example, hours of nursing time, number of physician visits, lost workdays, gained life-years)? | | Yes | No | No | Yes | Yes | Yes | No | No | No | No | Yes | No | Yes |
| 6. Were costs and consequences valued credibly? | | No | Yes | Yes | Yes | Yes | Yes | Yes | Yes | No | No | No | Yes | Yes |
| 7. Were costs and consequences adjusted for differential timing? | | Yes | Yes | Yes | No | Yes | Yes | Yes | Yes | No | Yes | Yes | Yes | Yes |
| 8. Was the incremental analysis of costs and consequences alternatives performed? | | No | Yes | No | Yes | Yes | Yes | No | No | No | No | Yes | Yes | Yes |
| 9. Was allowance made for uncertainty in the estimates of costs and consequences? | | Yes | No | Yes | Yes | Yes | Yes | Yes | Yes | No | Yes | Yes | Yes | Yes |
| 10. Did the presentation and discussion of study results include all issues of concern to users? | | No | No | No | No | No | Yes | No | Yes | No | Yes | Yes | Yes | Yes |
| No. of questions contemplated | Total | 5 | 4 | 6 | 8 | 8 | 10 | 5 | 7 | 2 | 5 | 8 | 9 | 9 |

**Table 4: Quality of selected studies**
