# Supplementary material for: Cost-effectiveness of mandatory folic acid fortification of flours in prevention of neural tube defects: A systematic review
Source: PLoS One. 2021 Oct 21;16(10):e0258488. doi: 10.1371/journal.pone.0258488 (PMC8530293; doi:10.1371/journal.pone.0258488)
Supplement: Attachment — Submitted filename: response to reviewers (4).docx [file pone.0258488.s004.docx]

Dear Editor,

We thank you for the opportunity to have our manuscript **PONE-D-21-10351**, entitled "Cost-effectiveness of mandatory folic acid fortification of flours in prevention of neural tube defects: a systematic review", evaluated for publication by Plos One. We are grateful to the reviewers for their time and effort. We are happy to implement the suggestions to improve the manuscript, as described below.

Yours sincerely,

The Authors

Table 1: Summary

| **Questions** | **Reviewer 1** | **Reviewer 2** |
| --- | --- | --- |
| Is the manuscript technically sound, and do the data support the conclusions? | Yes | Yes |
| Has the statistical analysis been performed appropriately and rigorously? | Yes | Yes |
| Have the authors made all data underlying the findings in their manuscript fully available? | Yes | Yes |
| Is the manuscript presented in an intelligible fashion and written in standard English? | No | Yes |
| Review Comments to the Author | Yes | Yes |
| Do you want your identity to be public for this peer review? | No | No |

**Reviewer 1, comment 1:** “In this systematic review, the authors review and summarize the cost-effectiveness of mandatory folic acid fortification of flours for preventing neural tube defects while additionally aiming to verify whether the folic acid dosage, cost compositions and quality of the economic studies included influences the cost effectiveness of the outcomes. 13 studies were identified that provide information enabling the authors to conclude that folic acid fortification is cost-effective in all but one of the countries included. A comparison between high and low-income countries was not feasible as no studies were identified in low-income countries. The findings of this study will be useful to promote the implementation of folic acid fortification to prevent neural tube defects globally in places where it is not currently implemented.

**Our response:** We would like to thank you for your consideration and time spent reviewing our manuscript.

**Reviewer 1, comment 2:** “Major comments: The introduction does not sufficiently articulate the context and rationale for this study. The authors should rewrite the introduction to better describe the current global context of folic acid fortification and what is known about its cost-effectiveness and what evidence gaps remain that this review will fill. While the final paragraph provides some specific reasons justifying for the review, these do not all appear to be supported by the preceding background information and its justification should be strengthened.”

**Our response:** Thank you for your comment. We rewrote the introduction to better describe our rational for summarizing new evidence about the cost-effectiveness on mandatory FA fortification. As we included several sentences, paragraphs, and new references, we have not included them in this response to review. However, the reviewers can see in the manuscript with track changes.

**Reviewer 1, comment 3:** “Major comments: The protocol appears to be technically sound; however, the presentation of the results (particularly Tables 1 and 2) could be improved to improve understanding and readability. Currently, there is some overlap in content (e.g. comparison strategies appears in both) and table 2 is very long with varying levels of details across the studies. The authors should review and revise the tables such that they provide a more concise summary of only the most important characteristics and include a similar level of detail across all studies.”

**Our response:** Thank you for you comment. Summarize the characteristics of the included studies is a challenging issue in every systematic review. However, your comments were very helpful to rethink our tables. Based on that, we included a new table just to describe the cost composition of the studies, which is a secondary objective of our systematic review. Instead of writing the same information about cost in every study, we used a cleaner version, using columns to represent cost items (please see below). Thus, Table 2 was substantially reduced. We also removed duplicate information, as suggested by the reviewer.

**Reviewer 1, comment 4:** “Major comments: The discussion should be revised to better articulate the overall findings of the review (as they relate to the aims of the paper, including not only the cost-effectiveness of folic acid fortification but also the verification of the influencing factors that were assessed) and the implications for folic acid fortification to prevent NTDs globally. In addition, a comparison to what is already known on cost-effectiveness appears to be missing and is important given past systematic reviews have been done on the subject (as mentioned in the introduction) and this would help to put these results in context and support the conclusions.
Finally, the conclusion raises research gaps that are not directly related to the study at hand. This should be rewritten to more strongly to summarize the findings, relevant research gaps, and what this means for the global community.”

**Our response:** Thank you for your comment. As suggested by the reviewer, we reworded the discussion. We included several sentences and paragraphs in order to compare our results with the literature.

**Reviewer 1, comment 5:** “Minor comments: 1. The manuscript would benefit from an overall review by a copy editor as some phrases are awkwardly worded and hard to understand.”

**Our response:** Thank you for your comment. As suggested by the author, we used an academic proofreading service by an English native speaker.

**Reviewer 1, comment 6:** “Minor comments: 2. Corn flour is often referred to as maize flour in many countries; therefore, it would be helpful to note that somewhere in the manuscript if in fact they are the same.”

**Our response:** Thank for you comment. We included this information in the abstract and introduction, indicating that corn flour is also known as maize flour, as suggested by the reviewer.

**Reviewer 1, comment 7:** “Minor comments: 3. The addition of a background statement at the start of the abstract before describing the aim of the study would be useful to provide context.”

**Our response:** Thank you for your comment. We include a background statement in the abstract, as suggested by the reviewer.

**Reviewer 1, comment 8:** “Minor comments: 4. The aim of the study in both the abstract and introduction should be revised to be more clearly and consistently worded as what is written in lines 84-85 varies from what is stated earlier in line 21-23 and line 79 about wanting to compare low vs. high income economies and now stating the comparison is mandatory vs. non mandatory fortification programs.”

**Our response:** Thank you for your comment. Our primary objective is to investigate whether the mandatory FA fortification of flours is cost-effective as compared to non-mandatory fortification. However, we also included our secondary objectives, which are to verify whether FA dosage, cost composition, and the quality of economic studies influence the cost-effectiveness of outcomes. We included the secondary objectives to comply with our rational of the introduction.

“We aimed to investigate whether mandatory folic acid (FA) fortification of flours is cost-effective as compared to non-mandatory fortification, and to verify whether FA dosage, cost composition, and the quality of economic studies influence the cost-effectiveness of outcomes.”

**Reviewer 1, comment 9:** “Minor comments: 5. The findings on factors that influence the cost-effectiveness of folic acid fortification should be added to the abstract as this was an aim of the study.”

**Our response:** Thank you for your comment. We included those results in the abstract.

**Reviewer 1, comment 10:** “Minor comments: 6. Line 85-86: this statement does not add anything and could be deleted.”

**Our response:** Thank you for your comment. We deleted this sentence, as suggested by the reviewer.

**Reviewer 1, comment 11:** “Minor comments: 7. Lines 144-145: the reference to table 3 should be in results, not methods.”

**Our response:** Thank you for your comment. We removed from the methods the reference to Table 3. We replaced by “in the format of tables”.

**Reviewer 1, comment 12:** “Minor comments: 8. Lines 166-168: sentence is not clearly written - what is meant by “epidemiologic database” and what “other countries” are referred to?”

**Our response:** Thank you for your comment. “Epidemiologic database” means surveillance systems or hospital sentinels. “Other country” refers to the U.S. We replaced this sentence for the one below:

“Some studies [25–28,30–35] used the difference in NTD estimates between the pre-post fortification collection baseline data for birth defects surveillance systems or hospital sentinels [28]. Three studies [23,24,29] used estimates of NTD reduction from the U.S.”

**Reviewer 1, comment 13:** “Minor comments: 9. Lines 174-175: abbreviations CEA/CUA/CBA need to be written out at first mention in the text.”

**Our response:** Thank you for your comment. We wrote out the abbreviations indicated by the reviewer.

“For the economic analysis, there was a combination of two or more types, such as cost-effectiveness/cost-utility analysis (CEA/CUA) [25,27,29,31,32,35] and cost-benefit/cost-utility/cost-effectiveness analysis (CBA/CUA/CEA) [34]. However, some studies presented only one type of analysis, such as CUA [30] or CBA [23,24,26,28,33]”

**Reviewer 1, comment 14:** “Minor comments: 10. In table 1: Use of the response “not clear” should be revised to “not included or does not report” where possible as this is not typically a standard assessment.”

**Our response:** Thank you for your comment. We replaced for “not reported”.

**Reviewer 1, comment 15:** “Minor comments: 11. In table 2: The final column “Author’s conclusions” should be renamed “Results” and should be a brief summary with similar level of detail across studies.”

**Our response:** Thank for you comment. We reformulated the summary to report similar level of detail across studies.

**Reviewer 1, comment 16:** “Minor comments: 12. Table 1 & 2: NI is not a standard abbreviation.”

**Our response:** Thank you for your comment. We replaced for “not reported”.

**Reviewer 1, comment 17:** “Minor comments: 13. Table 3: Folic acid contents column is repetitive of what is in Table 1”

**Our response:** Thank for you comment. We deleted the column “country”. However, we kept the column “FA dosage” since the cost-benefit ratio in influenced by it. Keeping both information (FA dosage and cost-benefit ratio) would improve the understand of the reader, we suppose.

**Reviewer 1, comment 18:** “Minor comments: 14. Table 4: This should be made supplementary material and the results briefly summarized in the text.”

**Our response:** Thank you for your comment. As suggested by the reviewer, we included the quality of studies in the Supplementary Material.

**Reviewer 1, comment 19:** “Minor comments: 15. Line 253: results for “mediated ratio of 18.5:1” - no information is provided on how this was calculated. if it is an average of all country results then details on this should be added to the methods section.”

**Our response:** Thank for your comment. We included this information in the method section, as suggested by the reviewer.

“We also provided a comparable measure of the economic benefit of the mandatory FA fortification, by means of the return of the investment. When the return of investment was not available in the included studies, we calculated it by dividing total cost averted attributed to mandatory FA fortification by the total cost of the mandatory FA fortification program. It means that for each monetary unit spent on the program, there would be a return of X monetary units.”

**Reviewer 2, comment 1:** “The study examined the cost-effectiveness of mandatory fortification of flours, compared to non-mandatory fortification, with folic acid through a systematic review of literature. Authors have identified all key papers on the topic and presented an adequate analysis answering their research question. The manuscript is well-written and presents policy makers important knowledge on fortification effectiveness while planning birth defects prevention programs in their country. Best and conservative scenarios of cost benefit presented in this paper also help in making a strong case for fortification.”

**Our response:** We would like to thank you for your consideration and time spent reviewing our manuscript.

**Reviewer 2, comment 2:** “Comments to Authors: Page 3: Lines 61-68. I do not think this paragraph adds to the paper. I suggest you either delete it or bring it up in the Discussion with following focus: The evidence on safety of folic acid is well established by many previous papers, and summarized very well by Field and Stover which you have already cited (Reference: Field MS, Stover PJ. Safety of folic acid. Ann N Y Acad Sci. 2018 Feb;1414(1):59-71. doi: 10.1111/nyas.13499). Presenting something that is not definitive distracts from your work. There is also no basis for upper limit for folic acid as written by Nick Wald and can be used instead of the information presented (Ref: Wald NJ, Morris JK, Blakemore C. Public health failure in the prevention of neural tube defects: time to abandon the tolerable upper intake level of folate. Public Health Rev. 2018 Jan 31;39:2).”

**Our response:** Thank for your comment. We agree with the reviewer that evidence on safety of folic acid is well-established in the literature. Moreover, any of the included studies in our review investigated safety or excessive FA consumption (UL> 1000 µg). Based on that, we deleted the paragraph on these issues, as suggested by the reviewer.

**Reviewer 2, comment 3:** “Can you add a recommendation for cost-benefit analysis for mandatory fortification much needed in low-income countries as this will be key in promoting fortification policies in high burden countries.”

**Our response:** Thank you for your comment. We reword the “implications for healthcare policies and systems” to accommodate the reviewer’s recommendations.

“Although the results obtained from our systematic review are from high and upper-middle income countries, nutritional deficiencies and their consequences occur at different levels among countries, particularly in low- and middle-income countries. Therefore, we believe that the implementation of mandatory FA fortification of flour or rice in low- and middle-income countries will have substantial health benefits and a good value for money for the population, since these countries have faced higher shortage of resources.”

“According to the 2020 Global nutrition Report [47], there is a need to establish synergy between public health and equity through strategies that improve the nutritional status of the entire population. This will reduce healthcare costs and save lives. Moreover, the Sustainable Development Goals (SDG) 2030 Agenda [48] is aligned with the focus on reducing inequalities among populations worldwide.”

“Mandatory fortification provides equity in different health systems worldwide. Prior studies [34,35] affirm that fortification improved equity compared to the pre-mandatory fortification policy (voluntary fortification and FA supplement). Mandatory FA fortification in flour provided a reduction in NTD rates (74%) among indigenous Australians and adolescent mothers (54%) [35]. Further, a Brazilian study [49] revealed a decreased prevalence of NTDs by 37.7% among the offspring of adolescent mothers.”
